# Potentials in Li-Ion Batteries Probed by Operando Ambient Pressure Photoelectron Spectroscopy

**DOI:** 10.1021/acsami.1c12465

**Published:** 2022-01-31

**Authors:** Ida Källquist, Tove Ericson, Fredrik Lindgren, Heyin Chen, Andrey Shavorskiy, Julia Maibach, Maria Hahlin

**Affiliations:** ^†^Department of Physics and Astronomy and ^‡^Department of Chemistry-Ångström, Uppsala University, 751 20 Uppsala, Sweden; § MAX IV Laboratory, Lund University, 225 94 Lund, Sweden; ∥ Institute for Applied Materials (IAM), Karlsruhe Institute of Technology (KIT), Hermann-von-Helmholtz-Platz 1, 76344 Eggenstein-Leopoldshafen, Germany

**Keywords:** Li-ion battery, electrochemistry, electrochemical potential, photoelectron spectroscopy, operando, ambient pressure photoelectron spectroscopy, solid/liquid interface

## Abstract

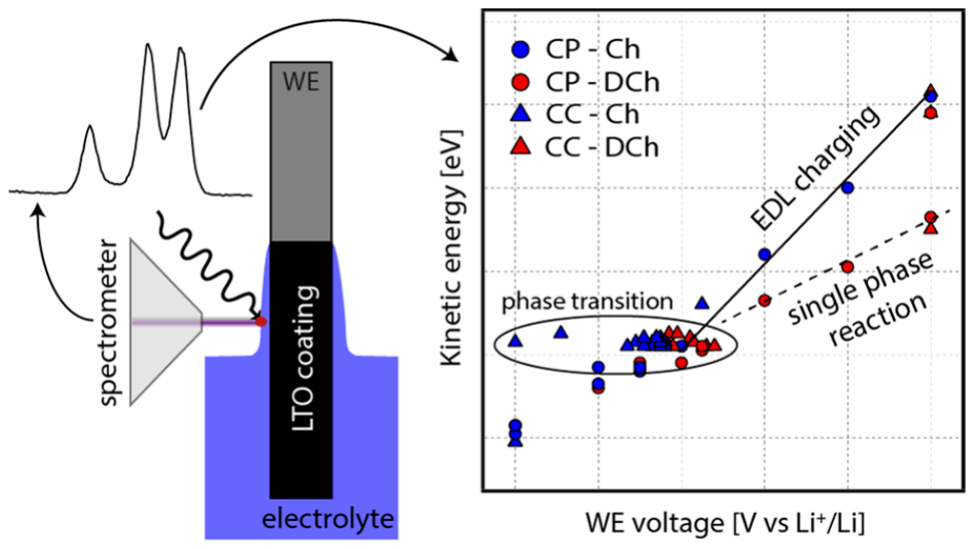

The important electrochemical processes in a battery happen at the solid/liquid interfaces. Operando ambient pressure photoelectron spectroscopy (APPES) is one tool to study these processes with chemical specificity. However, accessing this crucial interface and identifying the interface signal are not trivial. Therefore, we present a measurement setup, together with a suggested model, exemplifying how APPES can be used to probe potential differences over the electrode/electrolyte interface, even without direct access to the interface. Both the change in electron electrochemical potential over the solid/liquid interface, and the change in Li chemical potential of the working electrode (WE) surface at Li-ion equilibrium can be probed. Using a Li_4_Ti_5_O_12_ composite as a WE, our results show that the shifts in kinetic energy of the electrolyte measured by APPES can be correlated to the electrochemical reactions occurring at the WE/electrolyte interface. Different shifts in kinetic energy are seen depending on if a phase transition reaction occurs or if a single phase is lithiated. The developed methodology can be used to evaluate charge transfer over the WE/electrolyte interface as well as the lithiation/delithiation mechanism of the WE.

## Introduction

Photoelectron spectroscopy (PES) is one of the most used techniques to study interfaces in Li-ion batteries (LIBs) due to its surface and chemical sensitivity.^1,2^ While traditional ultrahigh vacuum PES has been limited to the study of solids, the development of ambient pressure photoelectron spectroscopy (APPES) instruments has diminished the vacuum constraint, enabling the study of both solid/gas and solid/liquid interfaces.^3−5^ With pressures up to ∼100 mbar in the analysis chamber, APPES can be used to study most organic electrolytes used in LIBs.^6−9^ Together with sample holders designed for electrochemical measurements, operando APPES measurements can be performed under conditions resembling those during real battery operation.^10−13^ By combining electrochemistry and photoelectron spectroscopy, both the chemical composition and the electrochemical potential differences can be probed. This gives valuable knowledge about important properties such as charge transfer, reaction pathways, and kinetics at the solid/liquid interfaces.

While solid/gas interfaces have been studied extensively during the last ∼15 years using APPES, solid/liquid interfaces remain challenging due to the short inelastic mean free path of the emitted photoelectrons in liquids and solids.^14−17^ To be able to probe the solid/liquid interface directly, either the solid or the liquid must be very thin (on the order of 10–30 nm, depending on the photon energy).^18^ Despite the limited thickness, the phases must remain functional to gain results representative of the system under study. Previous studies of solid/liquid interfaces in electrochemical systems have shown that in order for the liquid layer to be electrochemically active, the thickness of the electrolyte needs to be at least 10–20 nm.^19−21^ Due to this constraint, access to tender X-rays (∼4–6 keV) is essentially necessary to directly probe the solid/liquid interface of electrochemical systems operando.^18,19,21−23^

Unfortunately, APPES instruments with electrochemical measurement capabilities and tender X-rays are very rare. In this regard, it is interesting to consider what information can be gained by probing only the liquid electrolyte outside the electrode surface. This methodology is explored in this study. A three-electrode setup is used, where a voltage can be applied between the working and reference electrode (WE and RE, respectively) by the use of a potentiostat. Thus, the electron electrochemical potential of the WE (μ̅_e_^WE^) vs the RE can be controlled and/or measured. Further, by electrically connecting the WE to the spectrometer, their Fermi levels align, and μ̅_e_^WE^ can be used as an energy reference for the spectroscopic measurements. In this way, the kinetic energy (*E*_kin_) of the photoelectrons stemming from the liquid phase can be followed as a function of applied voltage to the WE.^19−21,24−26^

For an ideal polarizable interface (i.e., no charge transfer occurs), a shift in *E*_kin_ of 1 eV/V can be expected for the electrolyte peaks.^27^ This behavior, expected for pure electrical double layer (EDL) charging, has also been seen in many previous studies.^19,20,24,25,28,29^ However, studies performed during charge transfer are more scarce. In this case a 1 eV/V slope cannot generally be expected, as the equilibrium at the interface will be dominated by faradaic reactions rather than EDL charging.^27,30−32^ In our previous work, the behavior of a Au WE and a Cu WE was studied during charge transfer, and the results showed a deviation from the 1 eV/V shift.^33^ A model to explain this behavior was proposed based on equilibration of the Li-ion electrochemical potential at the WE/electrolyte interface.

In this work we further develop this model and methodology by investigating how changes in the different potentials μ̅ (electrochemical), ϕ (electrostatic), and μ (chemical) can be followed during LIB operation by operando APPES. In particular, we evaluate how the cycling protocol affects the potential differences between the WE and probed (bulk) electrolyte. Operando APPES measurements are performed on a 1 M LiClO_4_ in propylene carbonate (PC) electrolyte during electrochemical cycling of a Li_4_Ti_5_O_12_ (LTO) electrode. LTO was chosen as the WE due to its flat (main) lithiation plateau, corresponding to a first-order phase transition around 1.55 V vs Li^+^/Li.^34−36^ During this phase transition the chemical potential of LTO is constant,^37,38^ which facilitates the interpretation of the APPES results. In addition, LTO has a high power capability and relatively high reduction potential (above the onset of solid electrolyte interphase formation). These properties make LTO a suitable electrode material for our study. The presented measurements are of high importance to further understand the charge transfer kinetics at the electrode/electrolyte interface, as well as the phase transitions occurring in the active material during battery cycling.

## Methods

LTO based electrodes were prepared by mixing 80 wt % active material with 10 wt % sodium carboxymethyl cellulose and 10 wt % carbon black. Water was added to achieve a slurry that was mixed by ball milling for 1 h. The slurry was bar-coated onto an aluminum substrate and dried in a vacuum oven at 120 °C for at least 5 h. A LiNi_1/3_Mn_1/3_Co_1/3_O_2_ (NMC) slurry was prepared in the same manner, using the composition 80/10/10 wt % of NMC, poly(vinylidene fluoride-*co*-hexafluoropropylene), and carbon black, respectively. In this case *n*-methyl-2-pyrrolidone was added as the solvent. The NMC slurry was coated onto the same type of aluminum substrates as the LTO composite. The mass of active material was approximately 2.0 mg for LTO and 5.4 mg for NMC. A 1 M LiClO_4_ in PC electrolyte was prepared from as received LiClO_4_ and PC by mixing for several days.

Experimental capacities of the LTO and NMC electrodes were tested in pouch cells versus Li metal (see Figure S1 in the Supporting Information). The specific capacity was calculated to approximately 160 mAh/g for LTO and 130 mAh/g for NMC, in both cases based on the weight of active material. Since the NMC electrodes have a higher mass loading, these will have a large overcapacity compared to the LTO electrodes. This ensured that the LTO WE could be fully lithiated during battery operation.

**Figure 1:**
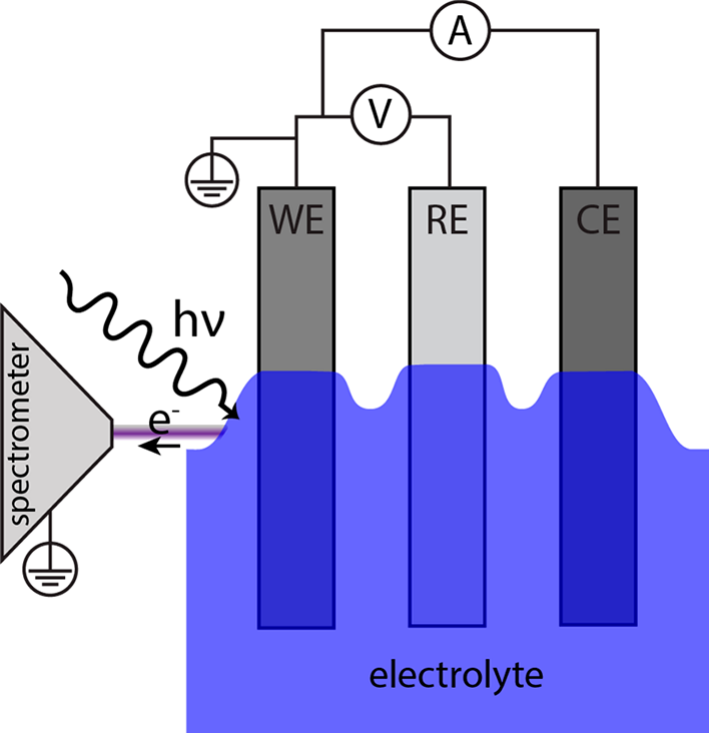
Illustration of the three-electrode setup used for operando APPES measurements. A thick part of the liquid electrolyte is probed.

Operando APPES measurements were performed at the HIPPIE beamline at MAX IV,^10^ using the electrochemistry end station. A three-electrode setup was used for the measurements. The setup is illustrated in Figure 1. The LTO composite was used as the WE, the NMC composite as the counter electrode (CE), and a Li metal piece as the RE, and the 1 M LiClO_4_ in PC solution was used as the electrolyte.

Electrochemical cycling was performed using a SP-200 Biologic potentiostat. The potentiostat was set on floating mode, and the WE was separately grounded to the same electrical ground as the spectrometer. Different cycling protocols were used, where either the WE voltage vs the Li/Li^+^ RE was controlled (constant potential (CP) mode) or the current between the WE and CE was controlled (constant current (CC) mode). For CC measurements the C-rate was based on the theoretical capacity of LTO (175 mAh/g) and the mass of active material (2 mg). Currents corresponding to C-rates between 1 C (350 mA = one charge in 1 h) and C/16 (22 mA = one charge in 16 h) were used.

For PES a photon energy of 1800 eV was used for all measurements. The incident angle of the photons was 55° relative to the sample normal, and the spot size on the sample was approximately 25 × 50 μm (vertical × horizontal). The spectra were recorded with a Scienta Hipp-3 analyzer. The photoemission was aligned with the sample normal, and the cone opening of the analyzer cone was 0.3 mm.

APPES measurements were performed on a liquid meniscus created by a dip-and-pull procedure.^19,33^ The electrodes were immersed (dipped) into the electrolyte so that the bottom edge of the sample plate was approximately 15 mm under the electrolyte surface. The dipped position was kept during electrochemical cycling, and the electrodes were only withdrawn (pulled) from the electrolyte beaker during APPES measurements. The two different electrode positions used during operando APPES are illustrated in Figure S2 in the Supporting Information.

For the APPES measurements the electrodes were retracted so that a sample spot previously immersed under the bulk electrolyte surface could be probed by APPES. To achieve this, the electrodes were pulled up by 3 mm (limited by the distance between the bulk electrolyte surface and analyzer cone opening). The electrochemical cell remained fully operational during APPES measurements as the major part of the electrodes was always kept in the electrolyte beaker. The liquid meniscus probed by APPES was thick enough to limit any signal from the solid electrode; i.e., the liquid thickness is larger than the probing depth (estimated to be ∼15 nm^33,39^). Between each spectroscopic measurement the electrodes were redipped to the same height. The sample was moved 0.1 mm sideways between each measurement to ensure a fresh measurement spot and avoid beam damage. After a voltage was applied, no electrode material could be detected on any spot on the sample, including the electrode material kept above the bulk electrolyte surface (also depicted in Figure S2 in the Supporting Information). This is expected to be caused by the porosity of the composite electrode, which for standard batteries is designed to optimize the wetting of the material.

C 1s and O 1s spectra of the electrolyte were measured during electrochemical cycling for different voltages and/or currents. No normalization or energy calibration was performed. Curve fitting of the C 1s spectra was done using Igor Pro (version 6.37). For each C 1s spectrum three peaks with an intensity ratio of 1:2:1 were assigned corresponding to the different carbon environments of the PC molecule. The energy difference between the carbonate peak and the hydrocarbon peak was fixed to 5.7 eV. Additional peaks necessary to accurately fit the measured data were assigned to adventitious carbons. All peaks were set to a FWHM of 0.2 eV for the Lorentzian part, and the Gaussian part was allowed to vary between a minimum of 0.9 eV and a maximum of 1.3 eV.

## Results and Discussion

Below we start by introducing some general concepts and relations necessary to interpret the operando APPES measurements. This is followed by a presentation of the operando APPES results, including the electrochemical and spectroscopic data. The results and their implications are thereafter discussed based on a suggested model for evaluating potential differences in LIB systems using operando APPES.

### Combining Electrochemistry and Spectroscopy—Basic Concepts and Relations

In this study, we use a previously developed operando APPES methodology for solid/liquid interfaces^19,21,24,33^ to systematically evaluate the effect different currents and/or overpotentials have on *E*_kin_ of the electrolyte peaks. To connect APPES and electrochemical measurements, the equality between the Fermi level, *E*_F,_ and the electron electrochemical potential, μ̅_e_, is recognized (if determined versus the same reference). The electrochemical potential can conceptually be divided into one contribution from the chemical potential and one contribution from the electrostatic potential, according to
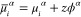
1where μ̅_*i*_^α^ is the electrochemical potential of species *i* in phase α, μ̅_*i*_^α^ is the chemical potential of species *i* in phase α, z is the unit charge (e.g., + 1, −1) and ϕ^α^ is the electrostatic potential of the phase α. The unit of μ_*i*_^α^ and μ̅_*i*_^α^ is here given in eV (rather than J/mol) to facilitate comparison to APPES measurements. For the reader unfamiliar with these electrochemical concepts, we recommend reading the excellent viewpoint by Boettcher et al.,^40^ which explains these concepts and the relationship between different potentials in electrochemistry.

Using operando APPES, the difference in μ̅_e_ between two phases can be probed by measuring one Fermi level relative to the other (e.g., by connecting one phase to the spectrometer and measuring *E*_F_ of the other). However, for nonmetals measuring *E*_F_ with (AP)PES is not straightforward, and it might be necessary to measure a core level instead. In this case a shift in the binding/kinetic energy of the core level may not directly represent the energy shift of *E*_F_. A shift in μ as a result of a change in chemical composition (e.g., during a redox reaction) will result in different chemical shifts for different core levels, depending on how different electrons and atoms are affected by the chemical reaction. This ambiguity can be avoided if the chemical composition of the measured phase (e.g., the electrolyte) can be kept constant, so that μ_e_^α^ is constant. In this case the core level shift is equal to the shift of *E*_F_ and only depends on the change in ϕ of the phase.

### Applicable Relations for Our APPES Setup

For our operando APPES measurements, a three-electrode setup with LTO as the WE, NMC as the CE, and Li metal as the RE is used. A 1 M LiClO_4_ in PC solution is used as the electrolyte. The chemical composition of the electrolyte can be regarded as constant since a large excess of Li-ions is present in the electrolyte. In addition, the Li-ions that are inserted (extracted) into the WE are also compensated by an extraction (insertion) of Li-ions at the CE (see further note S3a in the Supporting Information). When the chemical potential of the electrolyte is constant, it follows from eq 1 that Δμ̅_e_^el^ = −Δϕ^el^ (with *z* = −1 for an electron). This condition serves as a foundation for our suggested model, presented below.

In our setup, the WE is electrically connected to the spectrometer, and thus *E*_F_^WE^ serves as the energy reference in the APPES measurements (see also note S3b in the Supporting Information). In the electrochemical measurements, the Li/Li^+^ RE serves as a fixed reference with a constant electron electrochemical potential (note S3c in the Supporting Information). Since μ̅_e_^WE^ = *E*_F_^WE^ is continuously measured vs the RE during the operando APPES measurements, a kinetic energy measured vs *E*_F_^WE^ can also be referenced against the RE by adding the difference in electron electrochemical potential between the WE and the RE (i.e., −1×V).

Thus, for our setup the shift in kinetic energy (Δ*E*_kin_) of any electrolyte core level measured by APPES can be used to evaluate Δμ̅_e_^el^ (vs the RE) according to
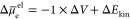
2where Δ*V* is the change in WE voltage (vs the RE), multiplied by −1 to gain the corresponding change in μ̅_e_^WE^ (see also Supporting Information note S3c). The derivation of this relationship and the necessary assumptions originate from our previous paper^28^ and are for convenience also explained in note S4a in the Supporting Information. Equation 2 shows how electrochemical measurements (providing Δ*V*) and APPES measurements (providing Δ*E*_kin_) can be combined to directly probe Δμ̅_e_^el^ (vs a fixed reference such as the RE). Equation 2 serves as the basis for the interpretation of the operando APPES results.

### Electrochemical Cycling of LTO

**Figure 2:**
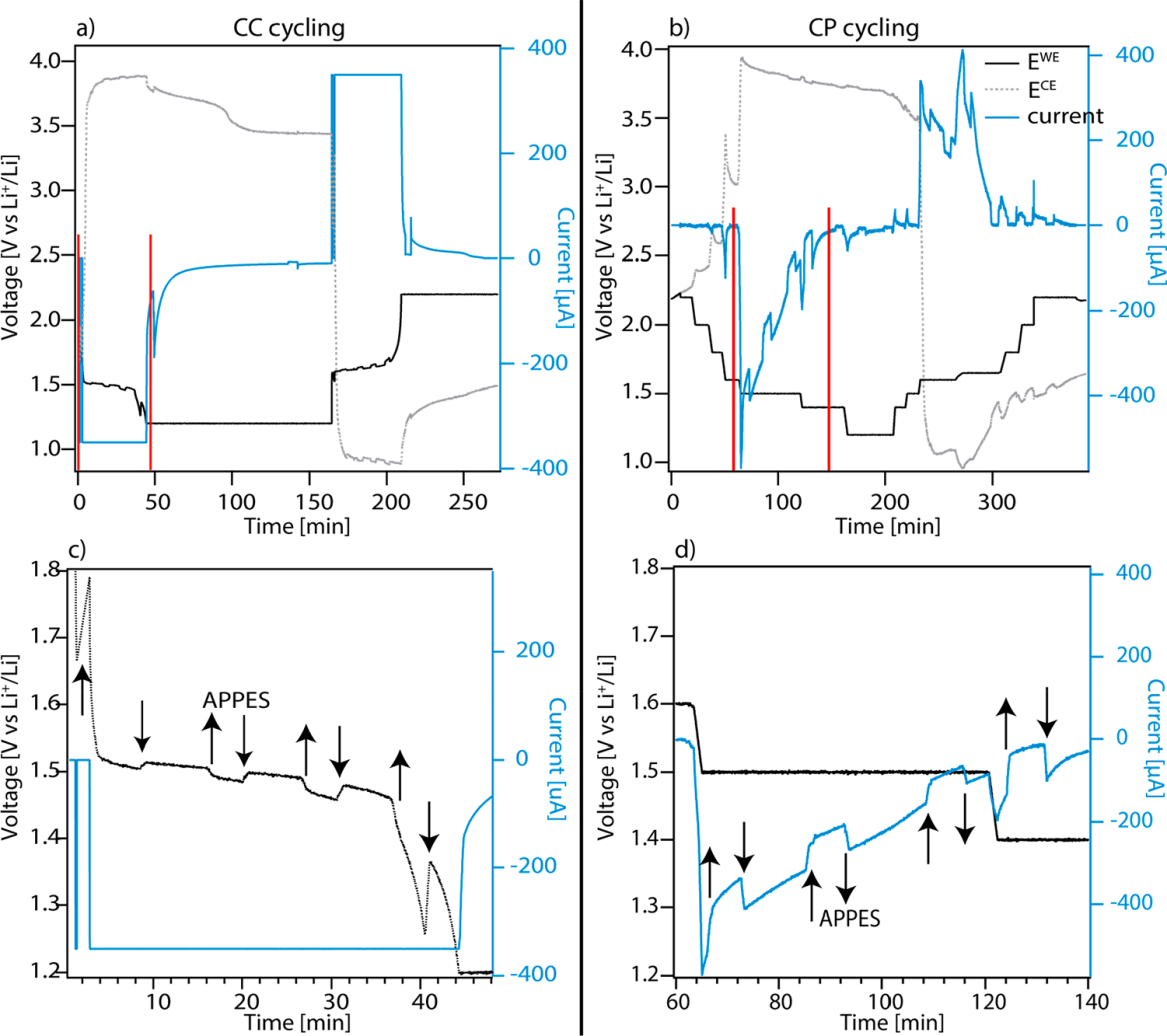
Electrochemical cycling of the three-electrode cell, using (a) constant current or (b) constant potential steps. (c) Zoom-in of the current and WE voltage in the time range marked with red lines in (a). (d) Zoom-in of the time range marked (b). The arrows indicate where the electrodes are retracted (arrow up) for APPES measurements and thereafter redipped (arrow down) for EC-cycling. The measurement time for each APPES measurement is approximately 5 min.

Figure 2 shows the electrochemical cycling using either constant current cycling (CC, Figure 2a and c) or constant potential cycling (CP, Figure 2b and d). The WE is cycled between 1.2 and 2.2 V vs the RE (all voltages are given versus the Li/Li^+^ RE in this work). For CC cycling, a potential hold is added at the cutoff voltages to allow for APPES measurements at the end points of charge/discharge. When the LIB is charged with a constant current the rate of the reaction is controlled, and the measured voltage corresponds to the overpotential necessary to support this reaction rate. Higher currents are thus expected to be correlated with a larger change in ϕ to increase the driving force for the reaction. When the LIB instead is charged/discharged by a series of constant potentials steps, the measured current corresponds to the rate of the reaction at a given applied voltage. If the voltage is above the onset of reduction (for charging), no faradaic reactions occur, and only a small capacitive current, corresponding to EDL charging, will be seen. The onset of a faradaic reaction during CP cycling is visible by a large increase in current. Depending on the applied voltage, the redox reaction can be studied either close to the equilibrium potential of the reaction or at a large overpotential.

In Figure 2a it can be seen that the WE (LTO) voltage curve exhibits a plateau around 1.5 V during charge (lithiation of LTO) and around 1.6 V during discharge (delithiation of LTO) for CC cycling using a C-rate of 1 C. Correspondingly, a large current is measured when the voltage is lowered to 1.5 V during charge and increased to 1.6 V during discharge for the CP cycling (Figure 2b). This is in accordance with the expected redox potential for the (de)lithiation reaction of LTO (i.e., Li_4_Ti_5_O_12_ + 3 Li^+^ + 3e^–^ ↔ Li_7_Ti_5_O_12_) at 1.55 V.^34,35^ At the lower cutoff voltage (1.2 V) the current never fully decays to zero, even after holding the voltage constant for ∼3 h (see Figure S3 in the Supporting Information). The measured current could correspond to a further lithiation of LTO toward Li_9_Ti_5_O_12_, i.e., full reduction of Ti^4+^ to Ti^3+^. A second reduction peak has previously been observed around 0.75 V for LTO, but a faradaic current can also be seen between the two reduction peaks,^41−43^ indicating that lithiation can continue at a low rate also between these voltages.

During cycling, the electrodes are regularly pulled up 3 mm for PES measurements, decreasing the area of the electrodes that are immersed in the bulk electrolyte. As can be seen in Figure 2, this gives rise to a larger polarization for the CC measurement (Figure 2c) and decreased currents for the CP measurement (Figure 2d). This shows that the rate of the redox reaction decreases when the electrodes are pulled up from the electrolyte beaker. This is expected since the ion transport at the top of the liquid meniscus is presumably limited.^20^ It can also be noted that the voltage of the NMC CE drops to very low values (around 1 V) during discharge. This is probably due to slow kinetics and resistances in the NMC composite, which was made thicker than LTO to ensure enough Li was present in the cell to fully lithiate the WE. However, the charge/discharge curves of LTO are comparable to cycling of LTO performed in standard coin/pouch cells using the same voltage range.^34,35^ Thus, it can be confirmed that the APPES electrochemical cell functions as a regular battery cell. The capacity obtained for LTO during operando APPES measurements can be calculated to approximately 140 mAh/g on both charge and discharge based on the mass of active material.

### Kinetic Energy Shifts of the Electrolyte APPES Peaks during Battery Cycling

**Figure 3:**
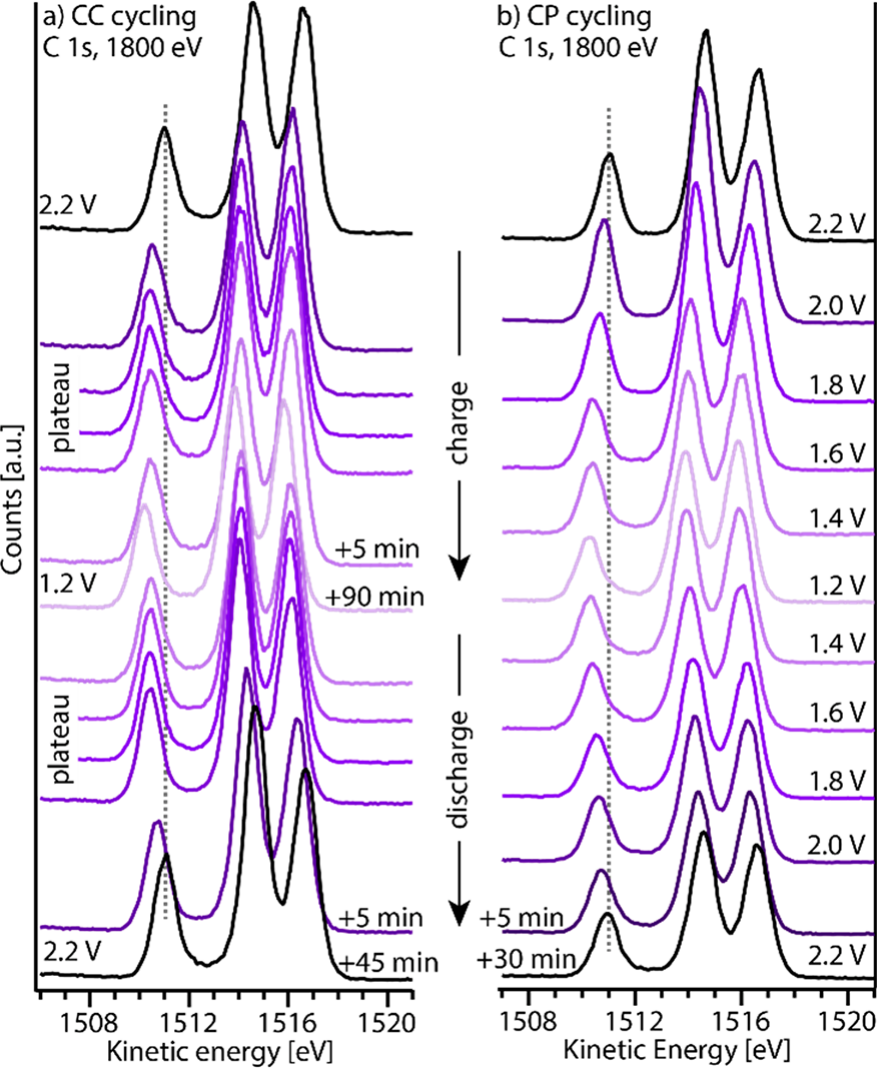
C 1s spectra measured during a full cycle of LTO using (a) CC cycling with a potential hold at 1.2 and 2.2 V and (b) using CP cycling. The color scale follows the voltage, where dark and light colors correspond to high and low voltage, respectively. A dashed line indicates the position of the PC carbonate peak at OCV (2.2 V).

To evaluate the changes in the electrochemical potential of the (thick) electrolyte, operando APPES measurements are performed during cycling of LTO. Both CC cycling and CP cycling are performed to evaluate if the cycling protocol affects the shifts in *E*_kin_ of the electrolyte photoelectron peaks. In Figure 3 an overview of C 1s spectra recorded during one full charge/discharge cycle of the LTO WE is shown. The spectra show the three peaks characteristic of the PC molecule (all spectra are shown in more detail together with curve fits in Figures S4–S7 in the Supporting Information).^7,38^Figure 3a shows spectra measured during CC cycling, using a C-rate of 1 C. Figure 3b shows spectra measured during CP cycling, using potential steps of 0.2 V between 2.2 and 1.2 V.

It is observed that the electrolyte photoelectron peaks move to lower *E*_kin_ when the WE voltage is lowered. When the WE voltage is increased, *E*_kin_ shifts back to its initial value. This is visualized by the dashed lines through the PC carbonate peak located at *E*_kin_ ∼ 1511 eV. The difference in *E*_kin_ between the upper and lower cutoff voltage is approximately 0.8 eV, i.e., somewhat lower than the total change in applied voltage. This can be seen for both cycling protocols. In more detail, it is interesting to note that *E*_kin_ seems rather constant when redox reactions are ongoing (i.e., during (de)lithiation). This is especially seen for the CC measurements. Additionally, it is noted that the shift in *E*_kin_ increases with time at the end points (1.2 and 2.2 V) as the current decays. At 1.2 V the shift in *E*_kin_ saturates after ∼30 min, as the current has reached a value below <10 μA (see Figure S3 in the Supporting Information). At 2.2 V the current decays to zero after ∼30–45 min (depending on cycling protocol), and *E*_kin_ returns to its initial value (see the black spectra in Figure 3).

### The Relationship between Kinetic Energy Shifts and Electrochemical Reactions in a LIB

For a LIB only ϕ of the electrodes will be changed before the onset of a (de)lithiation reaction. In this case, the electrodes will behave as ideal polarizable electrodes (no charge transfer), and a change of electrode voltage will result in the buildup of an EDL at the solid/liquid interface. This gives an electrostatic potential difference between the WE and the electrolyte, and a shift in the kinetic energy of the electrolyte APPES peak by 1 eV/V can be expected.

During (de)lithiation, charge transfer (of Li-ions) occurs over the electrode/electrolyte interface, and both ϕ and μ can be changed of both phases. In this case a shift in *E*_kin_ of 1 eV/V for the electrolyte photoelectron peaks cannot generally be expected, as the faradaic reactions will dominate the equilibrium at the interface.^27,30−32^ A deviation from a 1 eV/V slope was also seen in our previous measurements of Cu and Au during lithiation.^28^ In this section we seek to further understand the interfacial potential differences during charge transfer by evaluating the shifts in *E*_kin_ using different cycling conditions.

**Figure 4:**
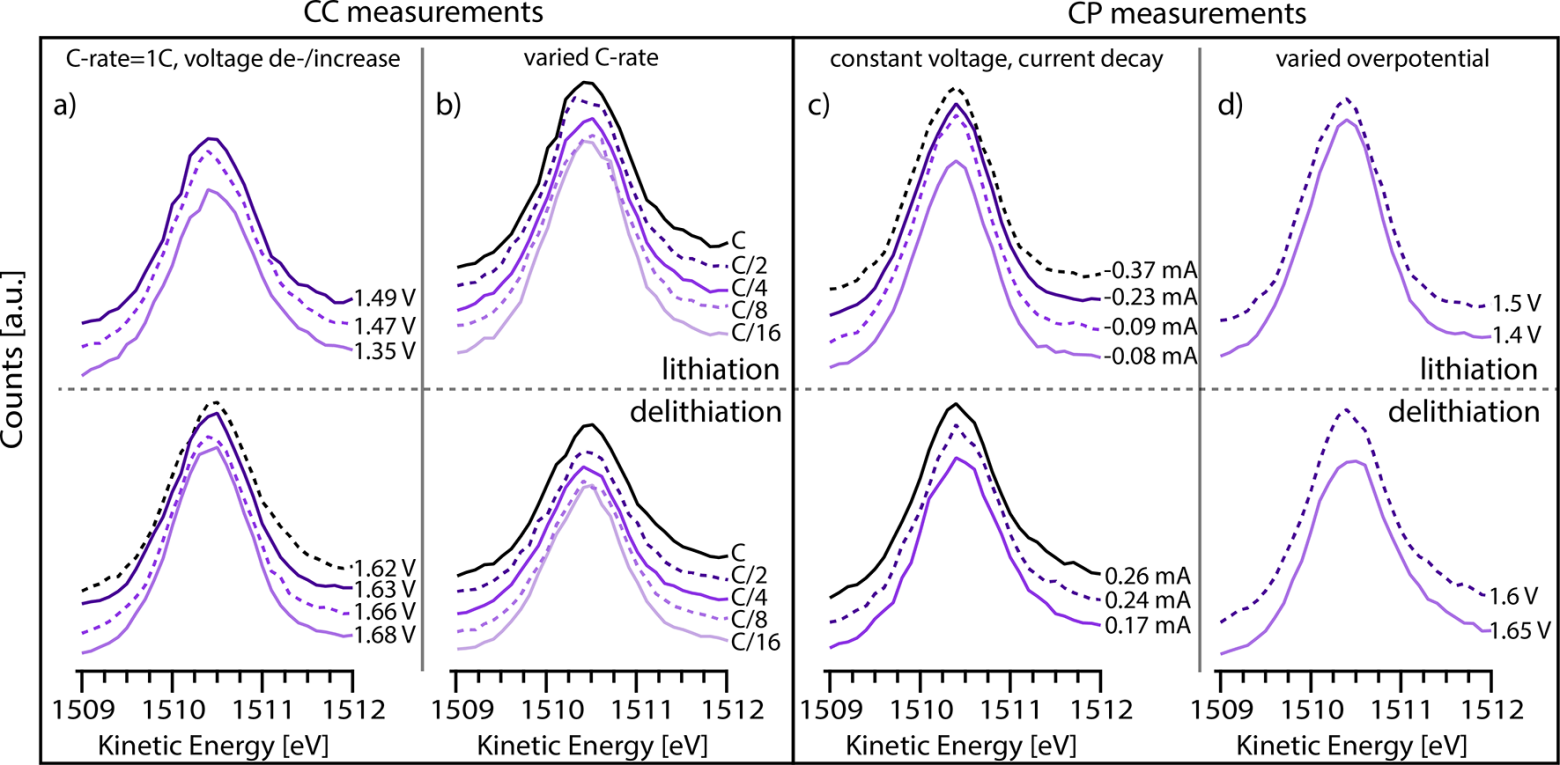
Kinetic energy of the carbonate peak in the PC solvent. The upper panel shows the results obtained during lithiation, and the lower panel the results obtained during delithiation. The different columns show CC cycling with (a) varying voltage or (b) varying current and CP cycling with (c) varying current or (d) varying voltage.

To investigate the deviation from the 1 eV/V shift in *E*_kin_ during (de)lithiation, additional measurements with different cycling conditions are performed on the (de)lithiation plateau. The aim is to evaluate if the shift in *E*_kin_ depends on the applied current and/or overpotential during (de)lithiation. Four different cases are evaluated during both lithiation and delithiation, as presented in Figure 4. In the first case (Figure 4a) the current is kept constant, and the effect of a change in voltage during cycling is evaluated. A high C-rate (1 C) is used since this gives rise to higher overpotentials and thus a larger variation in voltage during (de)lithiation. In the second case (Figure 4b) the effect of changing the constant current is evaluated. A total of five different C-rates are applied during the (de)lithiation plateau, ranging from 1 C to C/16. The APPES measurements are performed at the beginning of the (de)lithiation plateau (where the voltage is most stable) for all C-rates. However, due to polarization effects the voltage still varies slightly: from 1.51 to 1.56 V during lithiation and from 1.57 to 1.62 V during delithiation (the highest overpotential corresponds to the highest C-rate). Also, two cases using a CP protocol are evaluated. In Figure 4c measurements are performed during current decay after a change in applied voltage. In this case the effect of a change in current can be evaluate for a fixed WE voltage. Finally, different overpotentials are applied (Figure 4d) during (de)lithiation.

As can be seen from the results presented in Figure 4, none of these changes in current/voltage cause a notable change in *E*_kin_ of the electrolyte APPES peaks while LTO undergoes a phase transformation at the (de)lithiation plateau. This is a different behavior compared to the results of the relaxation *after* (de)lithiation, where it was seen that the peaks shifted with time as the current decayed (see Figure 2 and Figure S3 in the Supporting Information).

**Figure 5:**
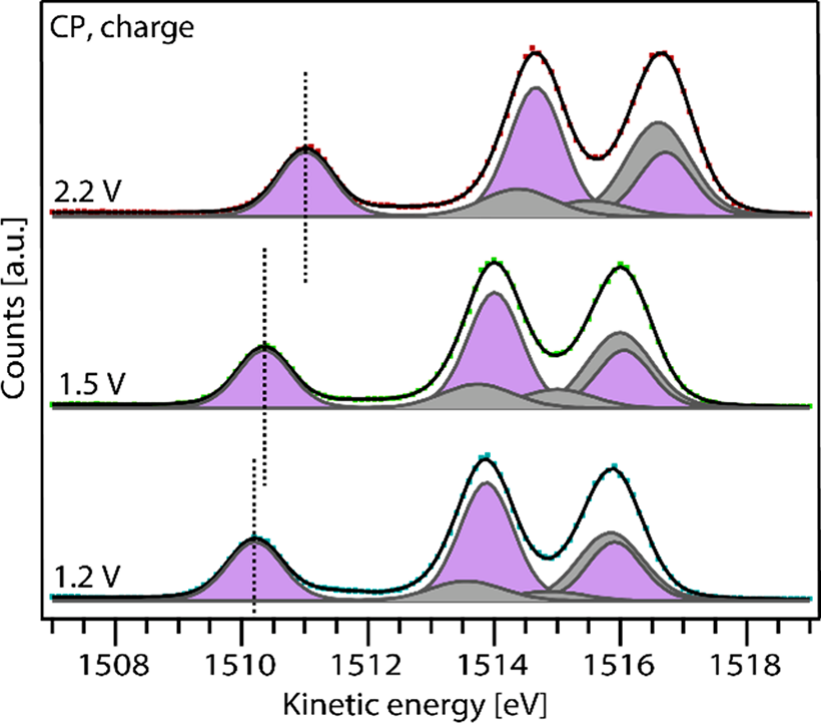
Curve fitted C 1s spectra measured at different constant potentials during charge. Dots show data points and solid lines the total curve fit. The vertical dashed lines indicate the kinetic energy of the PC carbonate peak.

To thoroughly evaluate the relation between shifts in *E*_kin_ and WE voltage during a complete charge/discharge cycle of LTO, all spectra presented in Figure 3 and 4 are curve fitted. A few examples are shown in Figure 5, while curve fits for all measurements are shown in Figures S4–S7 in the Supporting Information. Since the PC molecule consists of four carbon atoms in three different chemical environments, the C 1s spectra of pure PC will consist of three different C 1s peaks with an intensity ratio of 1:2:1.^7,44^ These are represented by purple peaks. In order to accurately fit the spectra, peaks corresponding to adventitious carbon bonded to hydrogen and/or oxygen are also needed (gray peaks). Such compounds are commonly seen for LIB electrolyte drops.^7,8,33^ From the curve fits it can be seen that the PC peak stemming from the carbonate group (marked with dashed lines in Figure 5) is the only peak that does not overlap with an adventitious carbon peak. Hence, this peak position is not affected by surface carbons and is exclusively representative of *E*_kin_ for the electrolyte solvent. The PC carbonate group is therefore used to track shifts in *E*_kin_ of the electrolyte solvent as a function of WE voltage.

**Figure 6:**
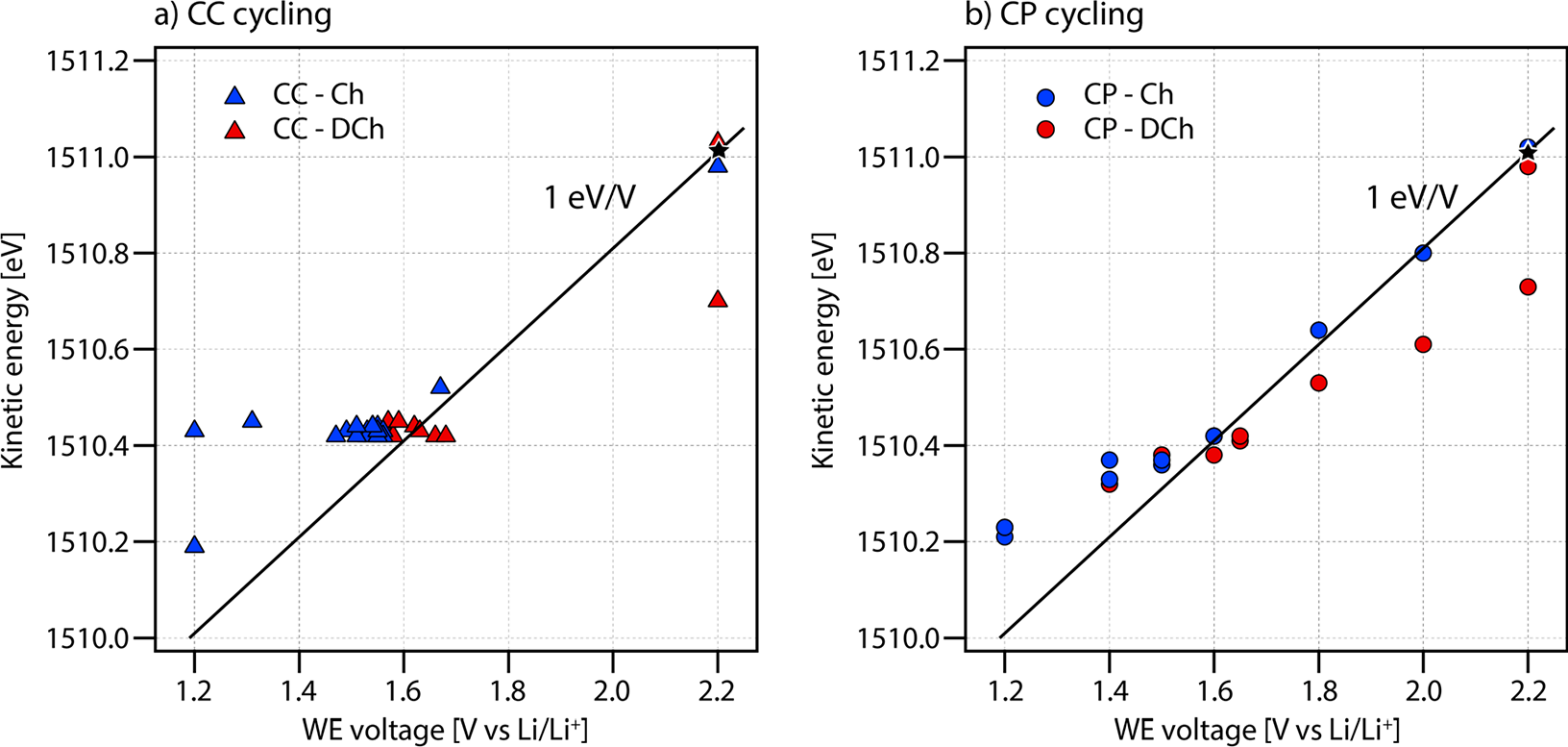
Kinetic energy of the PC carbonate peak as a function of WE voltage during (a) CC cycling and (b) CP cycling. Blue markers are measured during charge and red markers during discharge. A black line corresponding to a slope of 1 eV/V, starting from the OCV value (black star), is included as a guide. The error of E_kin_ is estimated from the standard deviation of the measurement points measured at the same potential and is included in the marker size (<0.01 eV).

In Figure 6, *E*_kin_ of the PC carbonate peak is shown as a function of WE voltage. A black line with slope 1 eV/V, starting from the kinetic energy measured at OCV (2.2 V), is included as a guide to the eye. This line corresponds to the expected behavior for pure EDL charging. Data points measured during charge and discharge are shown in blue and red, respectively. Considering first the measurements performed during CC cycling (Figure 6a), it can be seen that *E*_kin_ is essentially constant during the (de)lithiation plateau located around ∼1.55 V. For CC cycling, APPES measurements can in principle only be performed at the cutoff voltages and during the voltage plateau, as the voltage changes very rapidly when no redox reactions occur. However, it can still be seen that the difference in *E*_kin_ between OCV and the onset of lithiation roughly corresponds to a shift of 1 eV/V, since the black line passes close to the first points during lithiation (at 1.55 V). During and after lithiation (1.55–1.2 V), the measured shift in *E*_kin_ is smaller compared to the 1 eV/V line.

Looking at the data points from the CP cycling (Figure 6b), it is possible to get a better picture of the behavior over the full voltage region. At the beginning of the charge, before the onset of lithiation (voltage region 2.2–1.6 V), the ratio between the shift in *E*_kin_ and the applied WE voltage is close to 1 eV/V (i.e., measurement points follow the black line). This is expected for pure EDL charging.^19,20,24,25,28,29^ However, during and after lithiation, the shifts are clearly smaller than 1 eV/V. During discharge the shift in *E*_kin_ is initially roughly reversed. After the delithiation plateau, the shift in *E*_kin_ is significantly smaller compared to the same voltage region (1.8–2.2 V) during the charge. The different behavior in this voltage region is believed to be related to the delithiation reaction occurring during discharge, while only EDL charging occurs during charge. Possible explanations for the different behaviors are further discussed below.

### A Suggested Model to Explain the Shifts in Kinetic Energy during Charge Transfer of Li-Ions

The deviation from a 1 eV/V slope implies that when the WE voltage (i.e., μ̅_e_^WE^) is changed during charge transfer, μ̅_e_^el^ of the probed electrolyte also changes. The measured shift in μ̅_e_^el^ (vs the RE) can have several possible explanations, including an *iR*-drop over the electrolyte, a change in μ_e_^el^ (due to, e.g., ion concentration gradients or a reduction of the electrolyte), or a change of ϕ^el^ when charge transfer occurs at the WE/electrolyte interface.

If the deviation from a 1 eV/V slope was due to an *iR*-drop, the slope would primarily be affected by the current. In this case, a CC measurement should result in the same slope regardless of voltage. This is not the case, as seen in Figure 6a. In addition, a varying current during charge transfer (see Figure 4b and c) does not appear to affect *E*_kin_. Thus, an *iR*-drop cannot consistently explain the measured results.

If the shift in μ̅_e_^el^ was due to an ion concentration gradient in the electrolyte as a result of Li-ion (de)intercalation at the interface, the effect should be most prominent for large currents (i.e., high rate of reaction). However, for our system the reaction rate is expected to be limited by Li diffusion in the WE bulk, and the Li-ion concentration in the electrolyte would equilibrate quickly (see note S5 in the Supporting Information). The deviation from a 1 eV/V slope can neither be related to electrolyte reduction and the following change of the electrolyte composition, as this should occur at reduction potentials characteristic of the electrolyte (typically below 1 V^2,45^). This is not where the deviation from the 1 eV/V slope is observed. Thus, a change in μ_e_^el^ can also be disregarded as an explanation of the results.

Instead, a decreased slope of *E*_kin_ as a function of WE voltage is observed at the reduction potential of the WE, in both these and previous results.^33^ In this manner, the only plausible explanation we can suggest for the deviation from a 1 eV/V shift in *E*_kin_ is that ϕ^el^ is changed when charge transfer occurs at the WE/electrolyte interface.

In our previous study we proposed a model to understand the shifts in ϕ^el^, based on the equilibration of Li-ions over the WE/electrolyte interface.^33^ Based on the new results in this study, we further elaborate on this model and continue the discussion to also include the change of the Li chemical potential of the WE.

In a LIB, the electrolyte is an ion conductor but an electron insulator. In this manner, equilibration of electrons cannot be achieved at the electrode/electrolyte interface, but Li-ion equilibrium can be established during charge transfer (i.e., during (de)lithiation) if given sufficient time. In the case of Li-ion equilibrium between the WE surface and the probed electrolyte, μ̅_Li^*+*^_^WE^ = μ̅_Li^+^_^el^ holds.^27^ This can be assumed if the redox reaction is occurring at limiting current conditions, set by the bulk diffusion of Li in LTO.

The movement of Li-ions over the interface can cause changes in both chemical and electrostatic potential of both phases. However, for an electrolyte with a high Li-ion concentration and high Li-ion mobility, the electrolyte composition can be assumed constant (i.e., Δμ_e_^el^ = Δμ_Li^+^_^el^ = 0). In this case a change in electrochemical potential will only stem from a change in ϕ^el^, and Δμ̅_e_^el^ can be linked to Δμ̅_Li^*+*^_^el^ (see eq 1, where *z* = −1 for e^–^ and *z* = 1 for Li^+^):
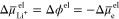
3From operando APPES, Δμ̅_e_^el^ can be measured (eq 2). Further, by using eq 2 in eq 3, we would in the case of Li-ion equilibrium (Δμ̅_Li^+^_^WE^ = Δμ_Li^+^_^el^) between the WE surface and the probed electrolyte get
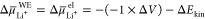
4From thermodynamics we can write the Li chemical potential as the sum of the electrochemical potential of the Li-ion and the electron, Δμ̅_Li_ = Δμ̅_Li_ = Δμ̅_Li^+^_ + Δμ̅_e_,^27^ and for Li-ion equilibrium between the WE surface and the probed electrolyte we arrive at (see also note S4b)
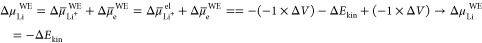
5As noted, eq 5 only holds for the assumption of Li-ion equilibrium. During nonequilibrium it is possible that |Δμ̅_Li^+^_^WE^| > | Δμ̅_Li^+^_^el^| since Δμ̅_Li^+^_^WE^ will change immediately upon a change in ϕ^WE^. Depending on the Li-ion mobility in the electrolyte, it may take some time for the electrolyte to respond to this change and re-establish equilibrium. However, this process is in our case expected to be fast due to the high Li^+^ concentration and high Li^+^ mobility in the electrolyte. Thus, it is reasonable to assume that practical equilibrium of the Li-ions at the WE/electrolyte interface can be achieved at limiting current conditions. In addition, even if Li-ion equilibrium is not achieved, the strive toward equilibrium would still be the driving force causing a change in ϕ^el^.

### Applying the Model to Interpret the Operando APPES Results of LTO

**Figure 7:**
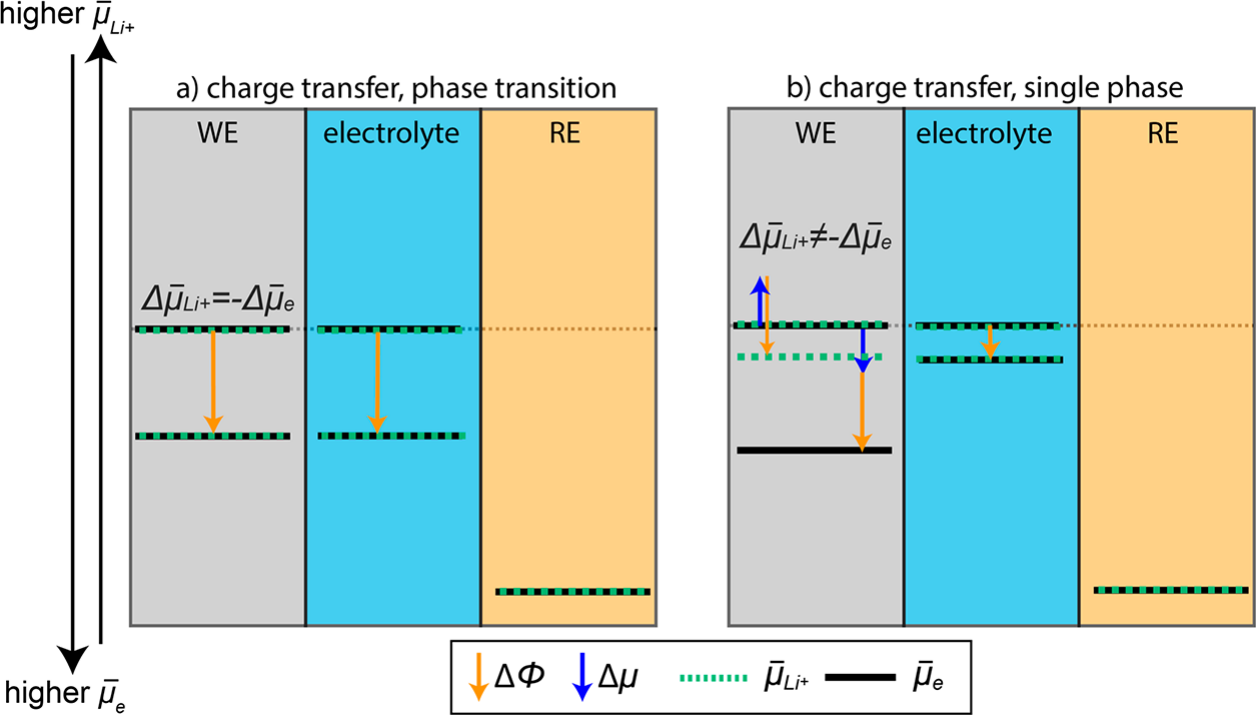
Schematic illustration of possible shifts in the electrochemical potential of Li^+^ and e when charge transfer/lithiation occurs during (a) a phase transition or (b) a single phase reaction. Shifts stemming from a change in ϕ are illustrated with orange arrows, while shifts stemming from a change in μ are illustrated with blue arrows. The shifts are illustrated with an arbitrary magnitude. During nonequilibrium it should be noted that μ̅_Li^+^_ may not be constant in the phases.

Relating the operando APPES results of LTO to the suggested model, eq 5 implies that if the measured *E*_kin_ of the electrolyte APPES peaks is constant, the Li chemical potential of the WE (μ_Li_^WE^) is also constant. This is seen for LTO at the (de)lithiation plateau and is in agreement with the thermodynamically predicted constant chemical potential during a first-order phase transition.^34,35,38^ During a first-order phase transition, μ^WE^ is constant for all species,^37,38^ and only ϕ^WE^ is changed when the WE voltage is changed during lithiation (by applying an overpotential). In this case the change in electrochemical potential of electrons and Li-ions is directly related through −μ̅_e_^WE^ = Δμ̅_Li^+^_^WE^ = Δϕ^WE^. To re-establish Li-ion equilibrium between the WE surface and the electrolyte when the WE voltage is changed, Δμ̅_Li^+^_^el^ = Δμ̅_Li^+^_^WE^ needs to be fulfilled. Since μ_Li^+^_ and μ_e_ of both phases are constant during phase equilibrium, this gives Δϕ^el^ = Δϕ^WE^. Consequently, Δμ̅_e_^WE^ and Δμ̅_e_^el^ will also be equal (since it is only affected by Δϕ). This is schematically illustrated in Figure 7a.

If also the chemical potential of the WE is changed during lithiation (e.g., during a single phase reaction), this contribution to μ̅^WE^ will counteract Δϕ^WE^ for Li^+^, while it will add to Δϕ^WE^ for the electron, due to the opposite charges. In this case, there is no direct correlation between Δμ̅_e_^WE^ and Δμ̅_Li^+^_^WE^. Consequently, the shift in Δμ̅_e_^el^ will *not* be equal to Δμ̅_e_^WE^. This case is illustrated in Figure 7b. It should be noted that the orange arrows (Δϕ) in the WE have the same magnitude, while the magnitude of the blue arrows (Δμ) for Li^+^ and e^–^ can be different. Depending on the ratio between Δμ and Δϕ, this will give a different slope of *E*_kin_ as a function of WE voltage.

The presented model can also be used to explain the different slopes seen in Figure 6 for charge/discharge based on the (de)lithiation mechanism. Since the Li-ions strive to establish equilibrium at the WE/electrolyte interface, Δμ̅_Li^+^_^el^ will be affected by Δμ̅_Li^+^_^WE^ at the WE surface. Any gradients in Δμ̅_Li^+^_^WE^ in the bulk WE will not be seen by APPES. During lithiation, the active material closest to the electrolyte will quickly undergo the phase transition from spinel Li_4_Ti_5_O_12_ to rock-salt Li_7_Ti_5_O_12_.^38^ After this, μ_Li_^WE^ remains constant at the WE surface, as the phase transformation continues into the bulk. In this region, *E*_kin_ is essentially constant (particularly visible during CC charging, see Figure 6a). The close to constant *E*_kin_ agrees with a phase equilibrium and a constant chemical potential of the WE according to eq 5. In this regard it can also be noted that the deviation of the applied/measured voltage from the standard equilibrium potential (1.55 V) directly represents the overpotential (η) of the reaction.

Below the first lithiation plateau (1.4–1.2 V), lithiation of the rock-salt phase can continue toward Li_9_Ti_5_O_12_. In this case μ_Li_^WE^ can change as more Li is intercalated into a single phase, giving rise to a shift in *E*_kin_ as the WE voltage is decreased. Assuming Li-ion equilibrium also in this region, the slope of ∼0.5 eV/V would signify that the change in applied voltage corresponds to both a change in overpotential and a change in Li chemical potential of the WE. The latter parameter can be estimated from eq 5, and the overpotential then corresponds to the deviation from the equilibrium potential at nonstandard conditions, according to the Nernst equation.^27^

During discharge the cycling is initially reversed, with lithium deintercalation from the rock-salt phase followed by the phase transition back from rock-salt to spinel. After the phase transition is completed, final delithiation of the spinel phase occurs. In this case, delithiation does not occur instantly but depends on the rate at which Li can diffuse out from the bulk of the WE. In this case a gradual change of Li concentration at the WE surface can be expected, which would correspond to a gradual change in μ_Li_^WE^ during discharge. This can explain the shifts in *E*_kin_ as measured by operando APPES in this region.

A similar explanation can be used for the behavior during relaxation at the cutoff voltages (1.2 and 2.2 V). As the applied voltage to the WE is kept constant, μ̅_e_^WE^ is constant. However, as the (de)lithiation becomes complete, μ_e_^WE^ can be changed followed by an equal change in ϕ^WE^ (in order to keep μ̅_e_^WE^ constant). Similarly, μ_Li^+^_^WE^ can be changed, and together with a change in ϕ^WE^ this will alter μ̅_Li^+^_^WE^. This in turn affects the Li-ion equilibrium between the WE and the electrolyte. Thus, the shift in *E*_kin_ seen during current relaxation at the cutoff voltages can also be explained by a change in μ_Li_^WE^. Alternatively, if (de)lithiation is fully completed and a redox couple no longer exists at the WE/electrolyte interface, a shift in *E*_kin_ can also be a result of the relaxation of Δϕ^el^ (driven by the Li-ion equilibrium) when charge transfer no longer occurs.

## Summary

**Figure 8:**
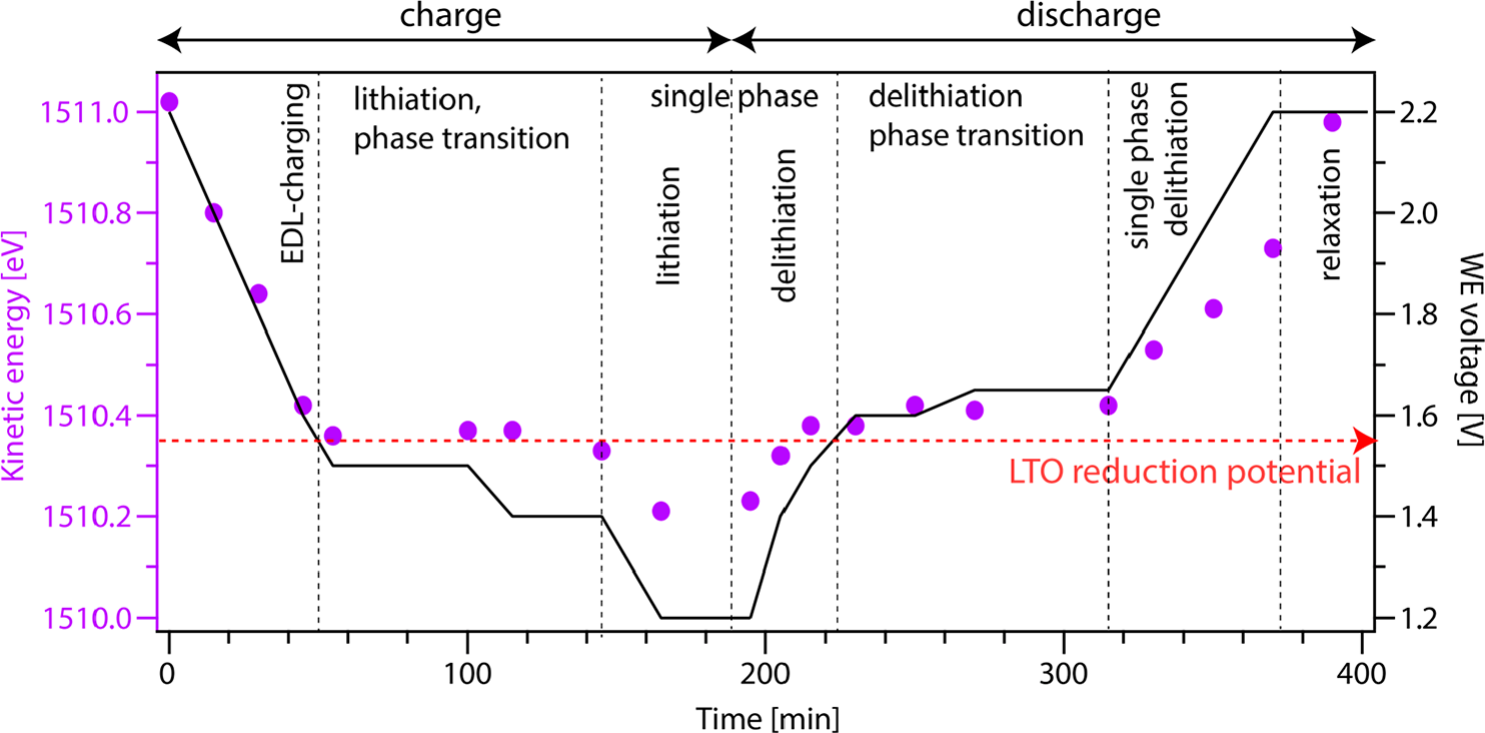
Kinetic energy of the PC carbonate peak (purple dots, left axis) and the LTO WE voltage (black line, right axis) as a function of measurement time. The LTO reduction potential (red dotted line) is added as a guide. The different processes occurring during charge/discharge are indicated by regions separated by black dashed lines.

In Figure 8 a summary of the results for the operando APPES measurements during a full charge–discharge cycle of LTO is shown. The figure displays the data points measured during constant potential cycling and shows the kinetic energy of the PC carbonate APPES peak (left axis) and the WE voltage (right axis) as a function of time. The cycling is initiated by EDL charging until the reduction potential of LTO is reached. In this first EDL region only ϕ^WE^ is changed, and the interface shows ideal polarizable behavior with a shift in *E*_kin_ of 1 eV/V. When the voltage is decreased below 1.55 V, lithiation begins, and LTO undergoes a phase transition (spinel to rock-salt). During the phase transition, the chemical potential of the WE remains constant, and a change in WE voltage during the phase transition will stem from a change in ϕ^WE^. During (Li-ion) charge transfer the Li-ions in the electrolyte strive to achieve Li-ion equilibrium with the WE surface and will thereby try to follow any changes in μ̅_Li^+^_^WE^. This gives an essentially constant *E*_kin_ in this region. After the phase transformation is completed, lithiation of the single rock-salt phase can continue. In this case both μ_e_^WE^ and ϕ^WE^ can be changed. The shift in *E*_kin_ measured in this region will depend on the relative magnitude of the shifts in μ_e_^WE^ and ϕ^WE^, as illustrated in Figure 7b. The total shift in *E*_kin_ during (de)lithiation can be used to estimate the shift in μ_Li_^WE^ according to eq 5. During discharge the rock-salt phase is first delithiated, followed by the phase transition back to the spinel. A similar behavior as during lithiation is seen here. After the phase transformation the final Li will gradually diffuse out through the WE surface, giving an additional single phase region. Finally, after the material is completely delithiated, a relaxation of the shift in ϕ^el^ back to its initial value is seen.

## Conclusion

In this work we have used operando APPES to probe a liquid LIB electrolyte during cycling of a LTO WE. The shift in *E*_kin_ of the (thick) electrolyte is measured versus the WE Fermi level. In this way the changes in the electron electrochemical potential difference between the probed electrolyte and the WE can be followed. Different cycling protocols are used to evaluate the effect of current and/or applied overpotential on the electron electrochemical potential difference. When no charge transfer occurs, the WE/electrolyte interface behaves as an ideal polarizable interface, and a shift in *E*_kin_ of the electrolyte APPES peaks of 1 eV per applied voltage is seen. However, during charge transfer the shift in *E*_kin_ deviates from the 1 eV/V slope. The results show that during the phase transition of LTO (around 1.55 V), *E*_kin_ of the probed electrolyte is very stable and does not change when the current or voltage is changed. At the end of charge/discharge of LTO, when instead a single phase is (de)lithiated, a shift in *E*_kin_ is seen, but it is significantly lower than 1 eV/V. We suggest a model to explain this based on the equilibration of Li-ions over the WE/electrolyte interface. Further, if the reaction rate is limited by Li diffusion in the bulk WE rather than Li-ion transport in the electrolyte, the shifts in *E*_kin_ of the electrolyte APPES peaks can be used to assess the change in Li chemical potential of the WE surface. Thus, by probing the electrolyte during charge transfer, the (de)lithiation mechanism of the WE can be studied. In this way, operando APPES can be a highly useful tool to gain further knowledge concerning the interfacial properties that influence the charge transfer kinetics and phase transitions occurring during cycling of LIBs.

## Supporting Information Available

Electrochemical cycling of pouch cells; schematic illustration of the APPES electrochemical cell; assumptions made for the experimental setup; measuring potential differences by APPES; relaxation of the current at 1.2 V; curve fits of APPES measurements; O 1s spectra (PDF)
