# Supplementary material for: Potentials in Li-Ion Batteries Probed by Operando Ambient Pressure Photoelectron Spectroscopy
Source: ACS Appl Mater Interfaces. 2022 Jan 31;14(5):6465–75. doi: 10.1021/acsami.1c12465 (PMC8832392; doi:10.1021/acsami.1c12465)
Supplement: Supplementary file 1 — am1c12465_si_001.pdf [file am1c12465_si_001.pdf]

Supporting information

Potentials in Li-ion batteries probed by operando ambient pressure photoelectron spectroscopy

Ida Källquist[a,*], Tove Ericson[b], Fredrik Lindgren[a,b], Heyin Chen[b], Andrey Shavorskiy[c], Julia Maibach[d], Maria Hahlin[a,b]

[a] Department of Physics and Astronomy, Uppsala University, 751 20 Uppsala, Sweden
[b] Department of Chemistry - Ångström, Uppsala University, 751 20 Uppsala, Sweden
[c] MAX IV Laboratory, Lund University, 225 94 Lund, Sweden
[d] Institute for Applied Materials (IAM), Karlsruhe Institute of Technology (KIT), Hermann-von-Helmholtz-Platz 1, 76344 Eggenstein-Leopoldshafen, Germany

*Corresponding author: ida.kallquist@physics.uu.se

Supporting note S1: electrochemical cycling of pouch cells

To evaluate the electrochemical performance of the LTO and NMC samples, one electrode from each batch was cycled in pouch cells prior to the beamtime. A half-cell setup where each electrode was cycled vs. Li metal was used. The same batch of electrolyte (1 M $LiClO_4$ in PC) as used for the APPES measurements was used for the pouch cells.

The two first full cycles for the LTO and NMC electrode are shown in Figure S1, using a constant current of +/- 0.1 mA. Both cycling curves show a behavior typical for LTO and NMC. The specific capacity can be calculated to approximately 160 mAh/g for both charge and discharge of LTO. For NMC the specific capacity is calculated to 140 mAh/g during charge and 120 mAh/g during discharge, indicating that the kinetics of the discharge (lithiation) process is slower compared to charge (delithiation) of NMC. Overall the specific capacity for NMC is somewhat lower compared to other experimental studies. This is probably due to the high mass loading of NMC resulting in rather thick electrode coatings that are not optimized for high specific capacities.

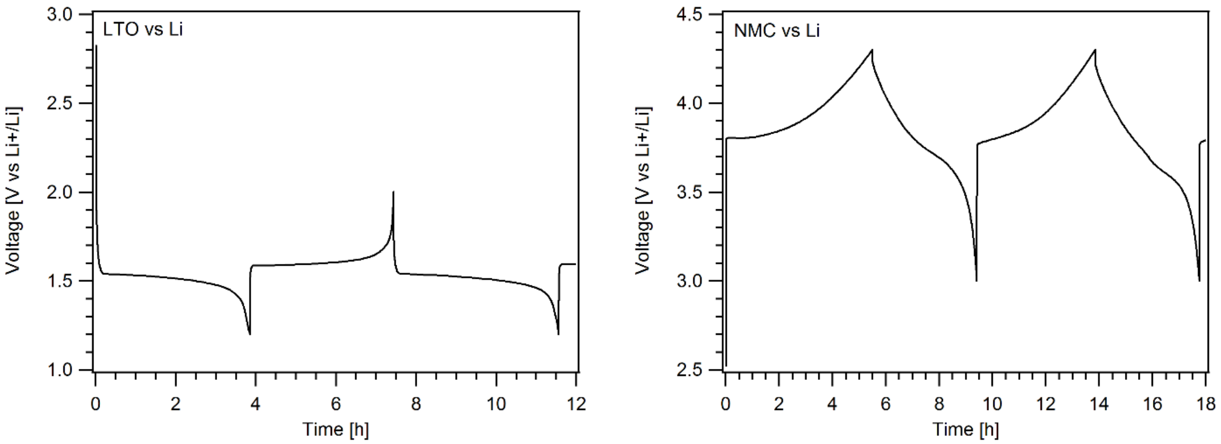

Figure S1: Cycling curves of LTO and NMC cycled in pouch cells vs. Li metal. A constant current of 0.1 mA is used for cycling of both cells.

For the operando APPES measurement performed at HIPPIE, pristine electrodes from the same batch were used. Comparing the electrochemical cycling from the APPES cell (see Figure 1 in main article) and pouch cells, the capacity is somewhat lower for the APPES cell (140 mAh/g vs. 160 mAh/g for LTO). During APPES measurements a decrease in current can also be seen when the electrodes are retracted, indicating that the reaction rate decreases when a larger part of the electrodes are above the bulk electrolyte surface. Judging from these results, it seems that the ion transport in the thin meniscus is limiting for the redox reactions rate for active material too far away (>3-5 mm) from the bulk electrolyte surface. To avoid effects from the slow ion transport in the meniscus the electrodes are re-dipped in between each APPES measurement.

Supporting note S2: Schematic illustration of APPES electrochemical cell

Figure S2 shows the two positions used for the WE during the operando APPES measurements. When electrochemical cycling is performed the WE is dipped so that 15 mm of the electrodes are below the bulk electrolyte surface. This is the maximum depth allowed by the beaker height. However, also in the spectra measured on the top edge of the LTO-coating (approximately 6 mm above the bulk electrolyte surface) a signal from Cl can be seen, and after applying a voltage no signal from Ti can be seen. This means that after applying a voltage the whole LTO composite is soaked with electrolyte and that the liquid layer is thicker than the probing depth.

The distance between the bulk electrolyte surface and analyzer cone opening (i.e. the APPES measurement spot) is ~3 mm. To be able to probe a spot that was previously under the bulk electrolyte surface, the electrodes are therefore retracted 3 mm from the electrolyte beaker for APPES measurements. The area of the electrolyte that is measured is marked with a red dot in both positions. In between each APPES measurement the electrodes are re-dipped to the EC cycling position.

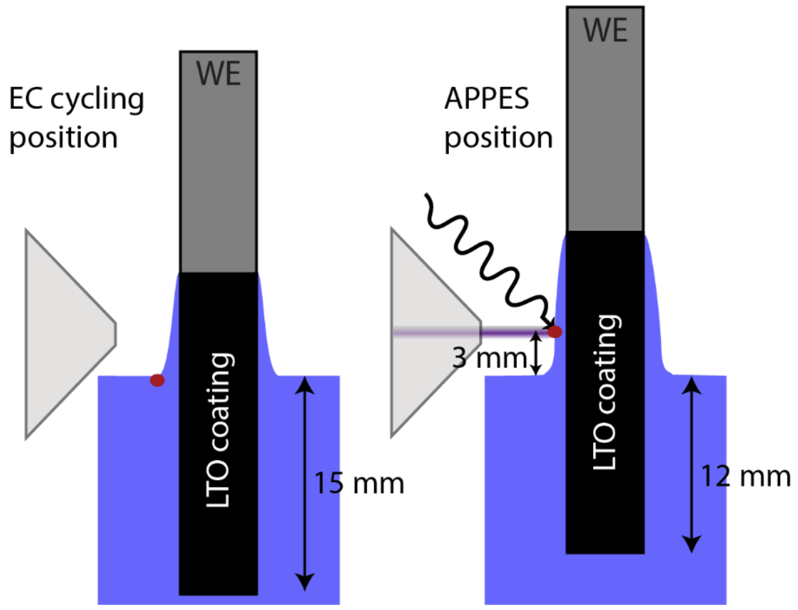

Figure S2: Schematic illustration of the APPES electrochemical cell setup, and the two positions used for the WE during electrochemical (EC) cycling and during APPES measurements.

Supporting note S3: Assumptions made for the experimental setup, and their implications

a) Constant composition/concentration of the bulk electrolyte

Due to the high Li-ion concentration and high Li-ion mobility in the electrolyte, any concentration gradients of Li-ions in the electrolyte will quickly be compensated by Li-ion movements. In addition, the Li-ion content in the electrolyte (volume ~10 ml, concentration 1 mol/l) is 0.01 mol. The total amount of Li that can be inserted into the WE (weight 2 mg) with a capacity of ~150 mAh/g, is approximately $1*10^{-5}$ mol. Thus, full lithiation of the WE (that also will be compensated by delithiation of the NMC CE) correspond to only 0.1 % of the Li-ions available in the electrolyte. Finally, the Li diffusion in the bulk WE is expected to be several orders of magnitude slower compared to the Li^+ diffusion in the electrolyte. Thus, the rate of the reaction would be limited by Li diffusion in the WE, and any gradients in Li/Li^+ concentration would thus only be expected in the WE. From these arguments we assume that the bulk electrolyte concentration remains constant throughout the measurements.

If the composition of the electrolyte is constant, also the chemical potential of the electrolyte is constant (since temperature and pressure is kept constant). This implies that $\Delta\mu_e^{el} = 0$ and $\Delta\mu_{Li^+}^{el} = 0$, and we get

$$\Delta\bar{\mu}_e^{el} = -\Delta\phi^{el} = \Delta\bar{\mu}_{Li^+}^{el} \, , \qquad \text{(Eq. S1)}$$

for our setup. Thus, since any change in electrochemical potential only stems from a change in electrostatic potential, all electron energy levels in the electrolyte will be shifted equally upon a change in potential. In this way any core level of the electrolyte can be measured to probe the change in Fermi level/electron electrochemical potential of the electrolyte.

b) Grounding of the WE to the spectrometer

By connecting the WE to the same electrical ground as the spectrometer, their Fermi levels will be aligned since both are electrical conductors.

This implies that all kinetic energies measured with APPES will be referenced versus the Fermi level of the WE, i.e vs the electron electrochemical potential of the WE, $\bar{\mu}_e^{WE}$.

c) Ideal non-polarizable behavior of the Li^+/Li reference

The potential of the Li metal will be governed by the reduction potential of the Li^+/Li redox couple (by definition, 0 V vs Li^+/Li). Since we have a very high concentration of both Li and Li^+ present at the RE/electrolyte interface, and only a minimal current is drawn through the RE (in order to measure the voltage), the electron electrochemical potential of the RE can be assumed constant throughout the APPES measurements.

This implies that any change in voltage measured between WE and RE solely stems from a change in electrochemical potential of the WE. Thus, $\Delta\bar{\mu}_e^{RE} = 0$ and $\Delta V = -(\Delta\bar{\mu}_e^{WE} - \Delta\bar{\mu}_e^{RE}) = -\Delta\bar{\mu}_e^{WE}$. Since the voltage measures the work required to move one unit positive charge between the electrodes, the voltage equals the negative of the electron electrochemical potential difference (in eV), or alternatively the voltage multiplied by -1 (e.g., -V) equals the work required to move one electron between the electrons in eV. The voltage multiplied by the unit charge of the electron has been used throughout the manuscript to relate the voltage to the electron electrochemical potential (in eV).

Supporting note S4: Measuring potential differences by APPES

a) When the assumptions made in note S3 holds, $\Delta\bar{\mu}_e^{el}$ can be measured by APPES in the following way:

When the chemical composition of the electrolyte (at the APPES measurement point) is constant, the measured shift in E_{kin} of any core level of the electrolyte correspond to the shift in electron electrochemical potential of the electrolyte (see note S3a). Since the WE is grounded to the spectrometer (see note S3b), the measured shift in E_{kin} is relative to the electron electrochemical potential of the WE. This gives:

$$\Delta E_{kin} = \Delta\bar{\mu}_e^{el} - \Delta\bar{\mu}_e^{WE}. \quad \text{(Eq. S2)}$$

Since $\Delta\bar{\mu}_e^{WE}$ is measured by the voltage (see note S3c), we have:

$$\Delta E_{kin} = \Delta\bar{\mu}_e^{el} + \Delta V \quad \text{(Eq. S3)}$$

and rearranging gives

$$\Delta\bar{\mu}_e^{el} = -\Delta V + \Delta E_{kin}, \quad \text{(Eq. S4)}$$

which is Eq. 2 that is presented in the manuscript.

b) When the assumptions made in note S3 holds, and in addition there is Li-ion equilibrium between the WE surface and the probed electrolyte, operando APPES can be used to follow the shift in Li chemical potential of the WE surface according to:

From thermodynamics we have

$$\Delta\mu_{Li}^{WE} = \Delta\bar{\mu}_{Li^+}^{WE} + \Delta\bar{\mu}_e^{WE}, \quad \text{(Eq. S5)}$$

and during Li-ion equilibrium $\bar{\mu}_{Li^+}^{WE} = \bar{\mu}_{Li^+}^{el}$, giving

$$\Delta\bar{\mu}_{Li^+}^{WE} + \Delta\bar{\mu}_e^{WE} = \Delta\bar{\mu}_{Li^+}^{el} + \Delta\bar{\mu}_e^{WE} \quad \text{(Eq. S6)}$$

Using $\Delta\bar{\mu}_{Li^+}^{el} = \Delta V - \Delta E_{kin}$ (Eq. 4 in manuscript) and $\Delta\bar{\mu}_e^{WE} = -\Delta V$ (from note S3c) this gives:

$$\Delta\bar{\mu}_{Li^+}^{el} + \Delta\bar{\mu}_e^{WE} = \Delta V - \Delta E_{kin} - \Delta V = -\Delta E_{kin} \quad \text{(Eq. S7)}$$

Combining these equations, we arrive at Eq. 5 in the manuscript:

$$\Delta\mu_{Li}^{WE} = -\Delta E_{kin} \quad \text{(Eq. S8)}$$

Supporting note S5: Relaxation of current at 1.2 V

To investigate the relaxation behavior at the lower cut-off voltage (1.2 V) repeated measurements are performed during the current decay at 1.2 V after CC charge, using a current of -200 µA. The results are presented in Figure S3. A reference spectrum from the lithiation plateau at 1.5 V is shown at the bottom of the graph. Initially no shift is seen in E_{kin} as the cut off voltage of 1.2 V is reached. As the current decays from -200 µA down to -10 µA, which takes around 30 minutes, a small shift in E_{kin} is seen. After this E_{kin} remains constant. The potential is kept at 1.2 V for a total of 2 h and 45 minutes but the current never fully decays to zero.

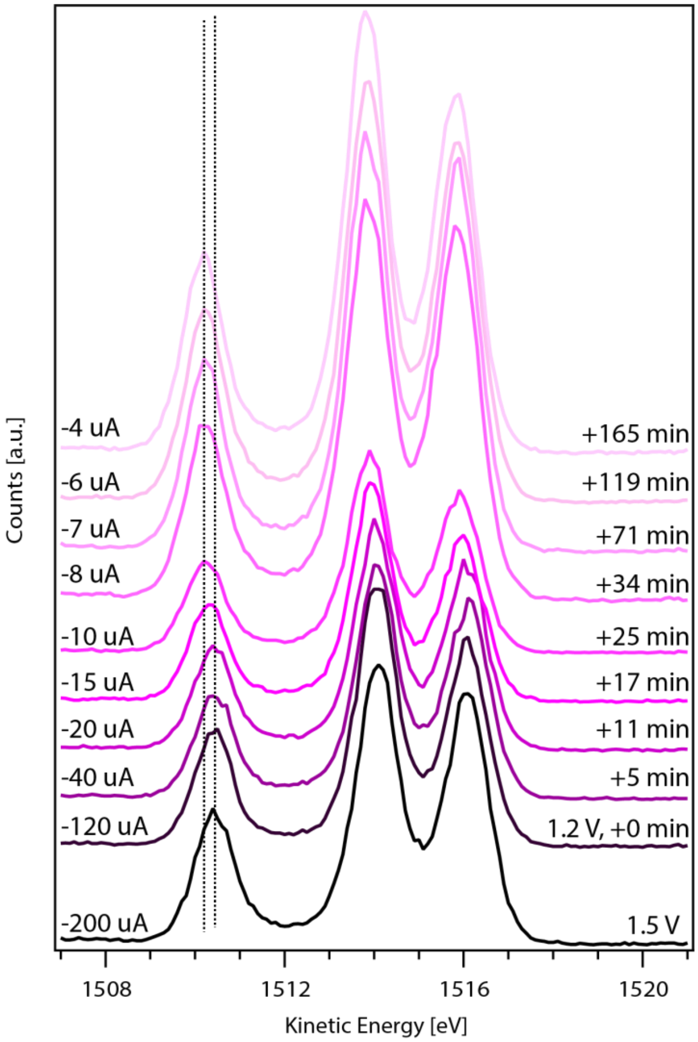

Figure S3: C 1s spectra measured on the electrolyte during relaxation of the current after the lower cut off voltage (1.2 V) is reached. The black spectrum shows a measurement performed on the lithiation plateau as a kinetic energy reference.

Supporting note S6: Curve fits of APPES measurements

In Figure S4-S7 the curve fits of the C 1s spectra are shown. The purple peaks correspond to intensity stemming from the PC molecule, and the grey peaks to adventitious carbon. As can be seen, the shape of the spectra is similar during all measurements. This indicates that the electrolyte is stable during the course of the measurement, and no substantial evaporation/degradation occurs. The shifts in kinetic energy are addressed in the main article.

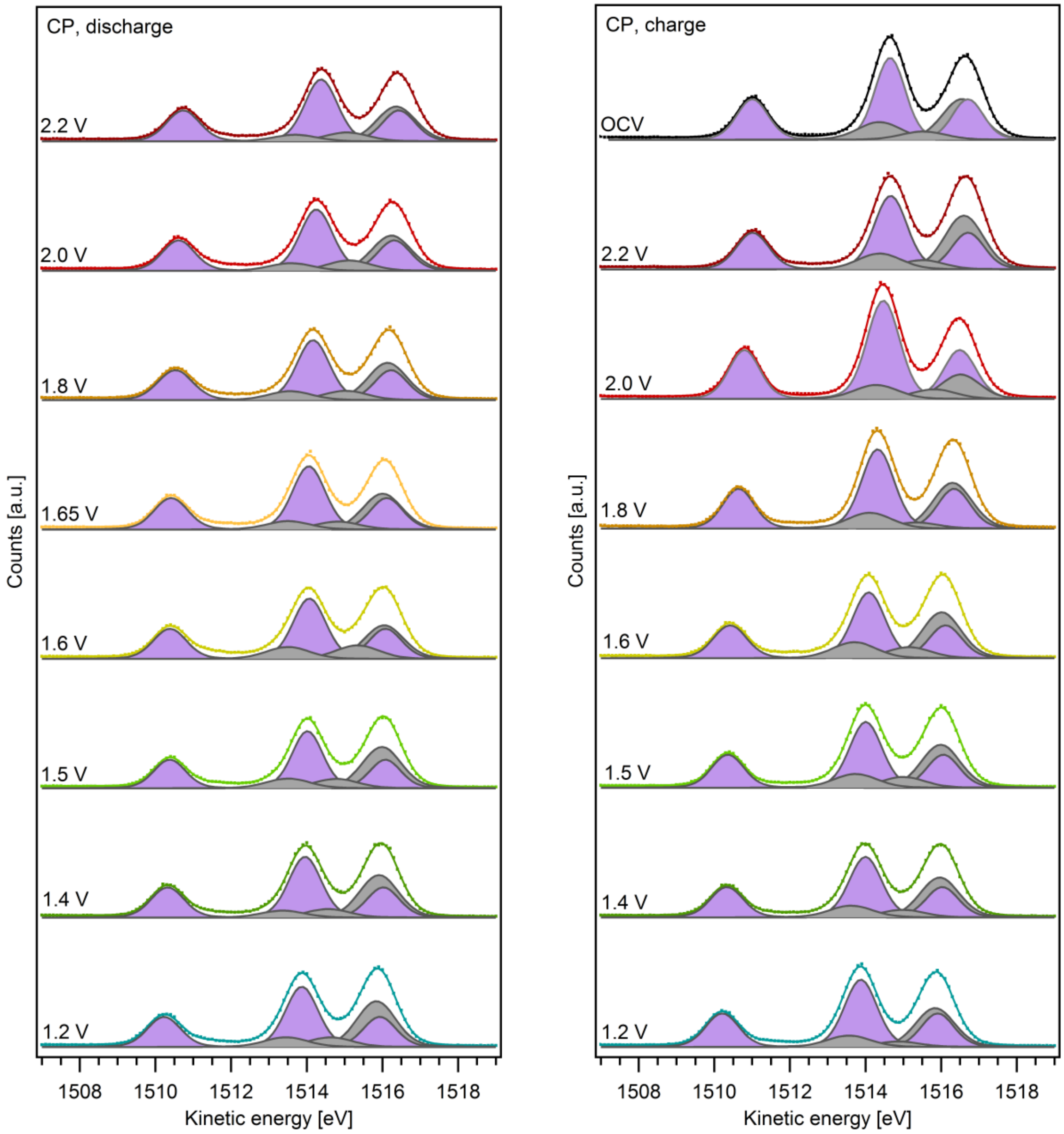

Figure S4: Curve fits of C 1s spectra measured at different constant potentials during charge and discharge of LTO in the voltage range 1.2 V to 2.2 V.

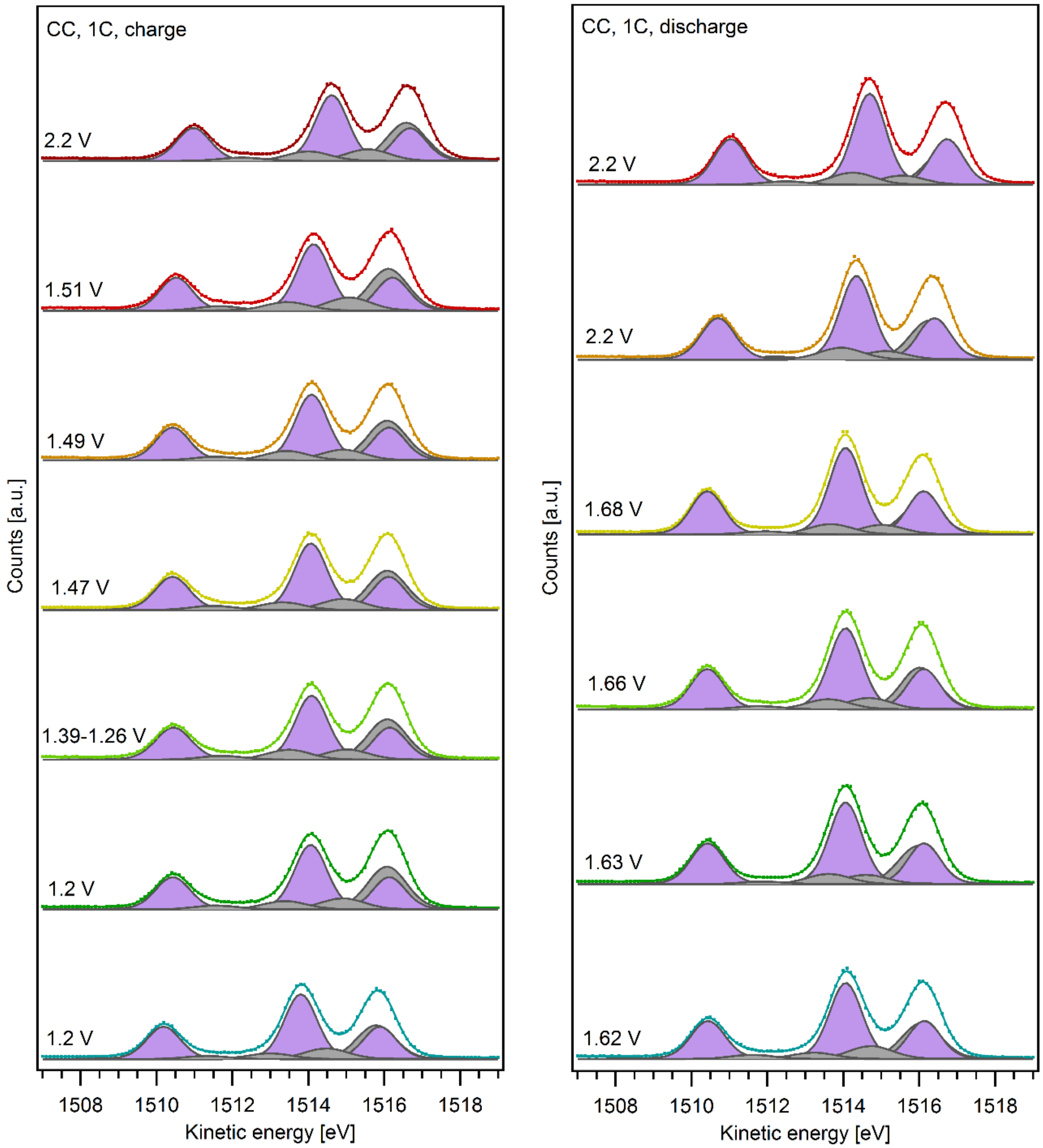

Figure S5: Curve fits of C 1s spectra measured during CC cycling (charge and discharge) using a C-rate of 1C.

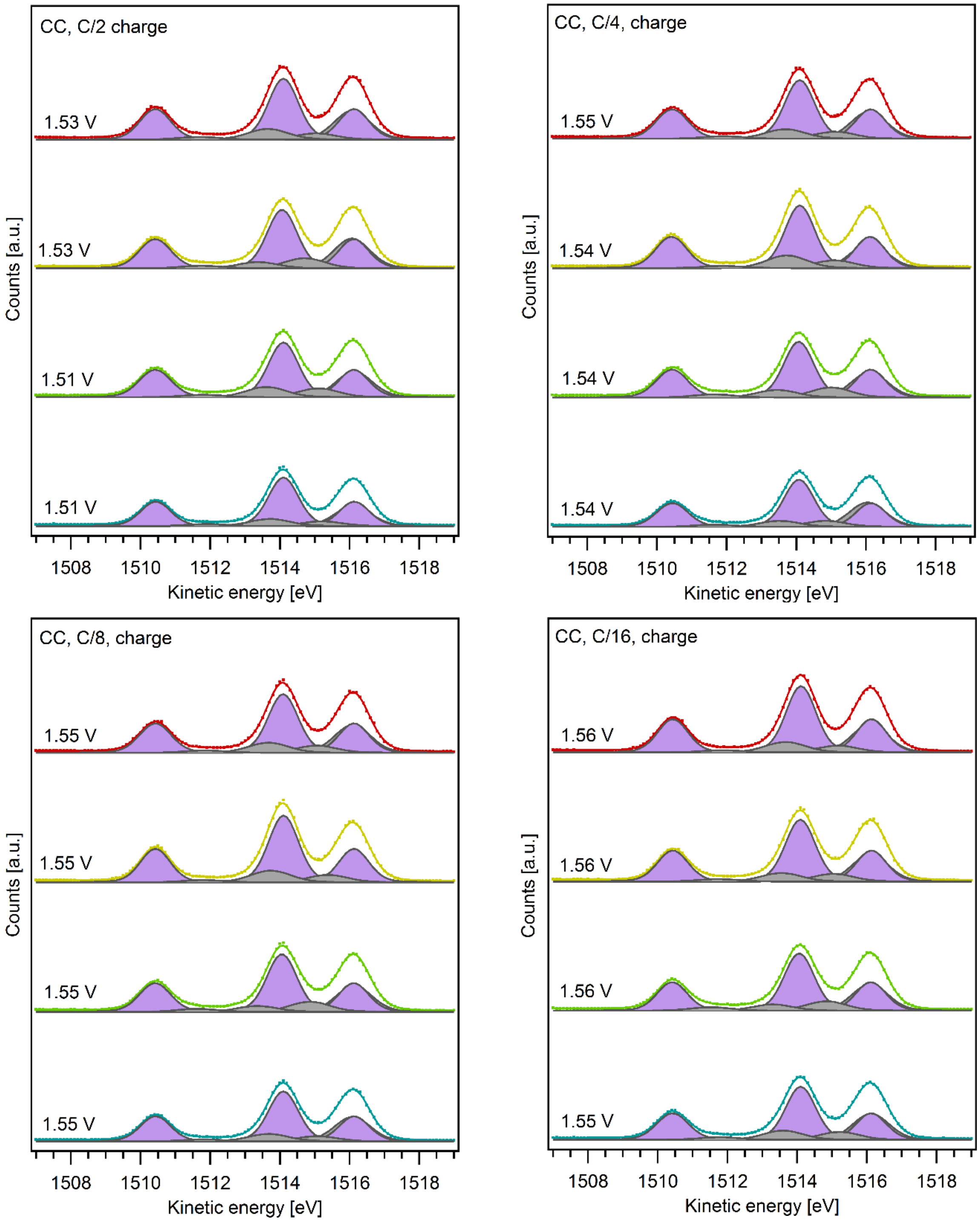

Figure S6: Curve fits of C 1s spectra measured during charge using different constant currents.

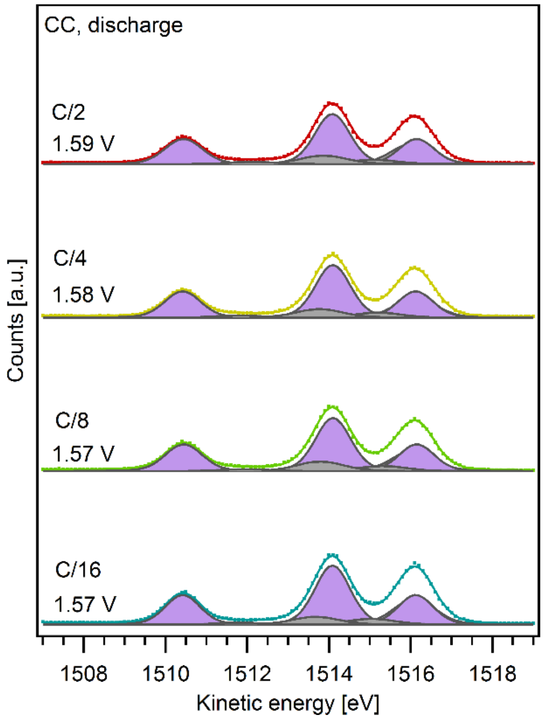

Figure S7: Curve fits of C 1s spectra measured during discharge using different constant currents.

Supporting note S7: O 1s spectra

In Figure S8, the O 1s spectra measured during CP cycling (left) and CC cycling (right) using a C-rate of 1 C are shown. The shape of the spectra is similar throughout the measurements, with some small variations in relative peak height that can be due to different amounts of C-O compounds on the surface, that may vary with voltage and measurement time. No metal oxide peak is visible (expected at ~1272 eV for the pristine LTO material), i.e., the electrode material is not visible in these measurements. In some spectra a tail at low KE is seen. This effect is not seen in the C 1s spectra, and is therefore attributed to salt degradation of the ClO_4-ion. The anions used in LIBs have previously been seen to be sensitive to beam damage [1, 2].

The kinetic energy shift between OCV (2.2 V) and the lower cut-off voltage (1.2 V) agrees well with the kinetic energy shift seen in the C 1s spectra. In both cases the total shift is approximately 0.8 eV/V.

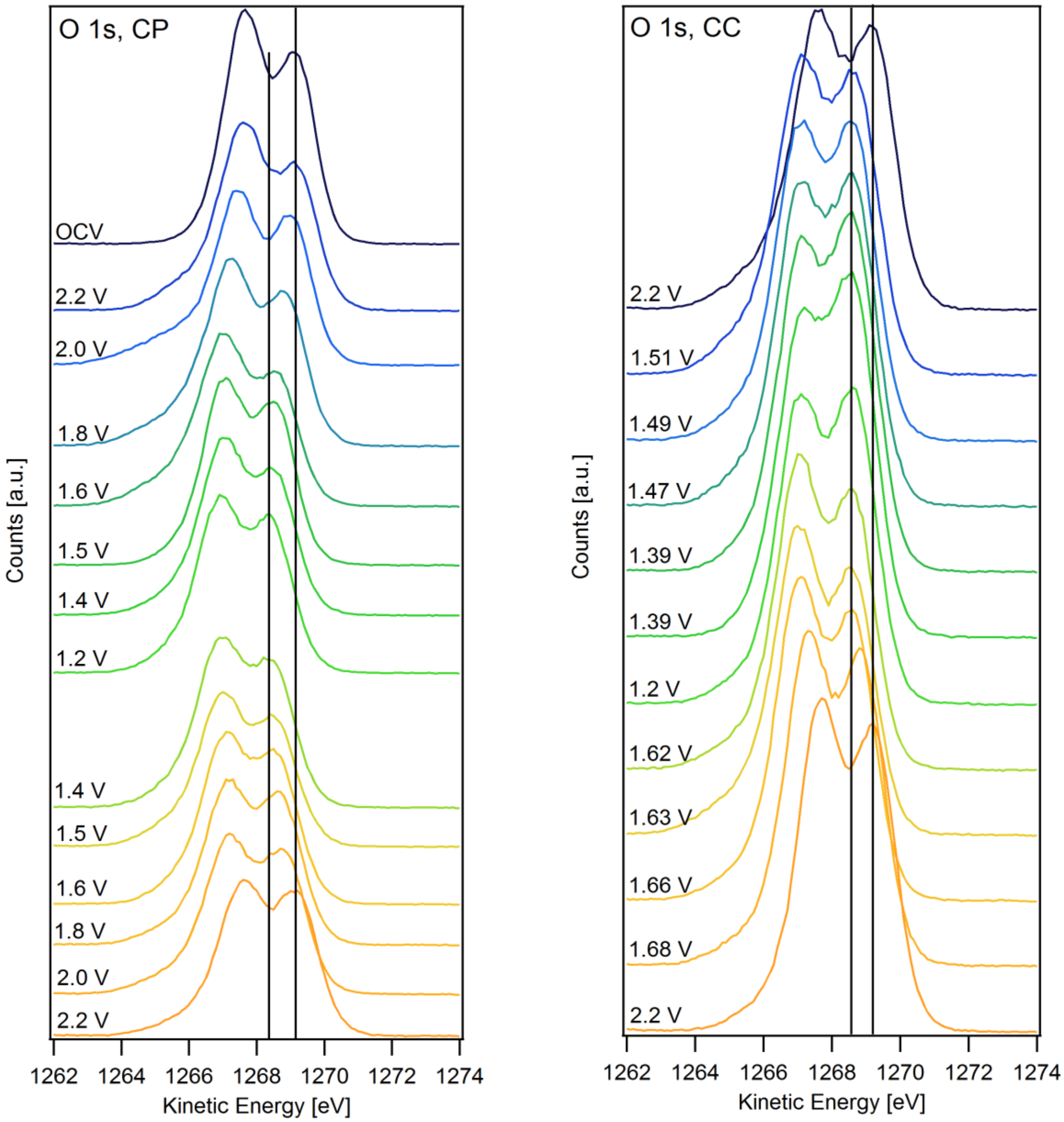

Figure S8: O 1s spectra measured at different voltages during CP cycling (left) and CC cycling (right).

Supporting references

[1] Younesi, R., Hahlin, M., and Edström, K., *Surface Characterization of the Carbon Cathode and the Lithium Anode of Li–O2 Batteries Using Liclo4 or Libob Salts.* ACS Applied Materials & Interfaces, 2013. **5**(4): p. 1333-1341.

[2] Maibach, J., et al., *Probing a Battery Electrolyte Drop with Ambient Pressure Photoelectron Spectroscopy.* Nature Communications, 2019. **10**(1): p. 3080.
